# Spatiotemporal interaction of tef head smudge disease (*Curvularia spp.*) and tef (*Eragrostis tef*) in the Western Amhara Region, Ethiopia, under the moderate (SSP245) and extreme (SSP285) climate change scenarios

**DOI:** 10.1371/journal.pone.0343054

**Published:** 2026-04-16

**Authors:** Melkamu Birhanie Mekonnen, Girmaye Dires Abeje, Mequannent Andualem Mekonnen

**Affiliations:** 1 Amhara Agricultural Research Institute, Adet Agricultural Research Center, Bahir Dar, Ethiopia; 2 State Key Laboratory of Ecological Safety and Sustainable Development in Arid Lands, Xinjiang Institute of Ecology and Geography, Chinese Academy of Sciences, Urumqi, China; University of Duhok, IRAQ

## Abstract

Tef is an important food security orphan crop in the Western Amhara Region, Ethiopia. However, its production is constrained by tef head smudge disease caused by *Curvularia spp*. Therefore, this study aims to model the spatiotemporal dynamics of tef head smudge disease and tef, as well as their spatiotemporal interaction. Therefore, this study conducted a comprehensive analysis of the current and projected geographic distribution of tef head smudge disease and tef by 2050 and 2070 under SSP245and SSP285 climate change scenarios using the MaxEnt model. The model has achieved 89.3% – 90.5% accuracy for tef and over 93% accuracy for tef head smudge disease across the current and future climate change scenarios. Tef is predicted to cover 33% of the Western Amhara region under the current climate scenario. However, its projections indicate shifts to 23.1% under SSP245 and 40.6% under SSP285 by 2050. By 2070, tef is projected to cover around 33.7% and 19.97% of the region under SSP245 and SSP285, respectively. Tef head smudge disease is predicted to occur on about 10,951 ha of land under the current climate change scenario. However, its distribution is predicted to be 6,361 ha and 18,812 ha by 2050 under SSP245 and 285, respectively. However, tef head smudge disease and tef are predicted to overlap on 9,659 ha of land under the current climate change scenario. This overlap is expected to increase to around 15,846 hectares (SSP285) by 2050, but decrease to 3,334 hectares (SSP285) by 2070. This study highlights the compounded challenges of climate change and disease pressure on tef production. Therefore, this research provides critical insights for policymakers and researchers to enhance resilience in tef cultivation and safeguard food security in the face of climate change.

## 1. Introduction

Tef (*Eragrostis tef*) is an underutilized orphan crop cultivated in Ethiopia for its edible grain [1]. It is cultivated across more than three million hectares of land, yielding around five million tonnes [2]. This precious orphan crop is a staple food for approximately 70 million people and supports the livelihoods of 6.5 million smallholder farmers in the country [2]. Tef accounts for 30% of total grain production in Ethiopia due to its adaptability to a wide range of environmental conditions [3,4]. It performs well at an altitude of 1700–2200 meters above sea level (m.a.s.l.), with an annual rainfall of 750–850 mm, and a temperature range of 10°C to 27°C [5]. The environmental conditions of the Western Amhara Region are suitable for tef cultivation and take the lion’s share in terms of area coverage and volume of production [6].

Tef is rich in several nutrients, and its nutritional profile is unparalleled by other cereals. It is rich in dietary fiber, protein, calcium, and iron [7]. Moreover, tef is gluten-free, and its gluten-free nature makes it ideal for individuals with celiac disease or gluten intolerance [8,1]. Tef grain has a remarkable shelf life, retaining its quality for over a decade without being damaged by pests such as weevils [7]. These distinctive nutritional and agronomic attributes of tef (*Eragrostis tef*) have catalyzed its demand worldwide. Currently, tef cultivation has expanded beyond its native region and is now grown in diverse geographic areas, including the United States of America, Canada, Europe, and Israel [9]. However, the production of this nutrient-rich, gluten-free food security crop is constrained by tef head smudge disease (*Curvularia spp.*) [9]

Tef head smudge disease, caused by *Curvularia spp*., is a devastating fungal pathogen affecting tef (*Eragrostis tef*) in warm, humid regions of Ethiopia. The occurrence of this disease on tef was documented in 1966 [10]. Since then, this disease has occurred sporadically at varying levels of disease incidence and severity. It was considered a minor pest on tef in major tef-growing areas of the Western Amhara region, Ethiopia. However, recent studies indicate a concerning shift in its epidemiological significance [11,6]. This disease has become prevalent and a major production constraint in Ethiopia in general and in the Western Amhara region in particular [10]. According to Mekonnen et al. [12], tef head smudge disease causes up to 62% yield loss on tef. Moreover, it significantly affects germination percentage, germination rate, seedling root length, seedling height, and vigor index [12]. Nowadays, tef head smudge is an important plant disease in major tef-growing areas, particularly in warm, humid areas of Ethiopia [11,12]. This disease is prevalent at the maturity stage, in mid-altitude areas, and improved varieties under a conducive environment [11,6]. This shift in tef head smudge disease from being a minor pest to a major pest could be due to climate change [13,14].

Climate change is a long-term shift in the average temperature, precipitation, and other aspects of the Earth’s climate system [15]. The long-term shift in the Earth’s climate system leads to rising temperatures, shifts in rainfall patterns, and an increase in the frequency of extreme weather events [16]. According to Lee et al. [17], the global average temperature has increased by 1.5 °C since the 19th century, and it is projected to increase by 1.5–2°C by the end of the 21st century. However, the global average temperature could be projected by 2.5–3°C under a moderate (SS245) climate change scenario, where there is a moderate inequality and uneven climate policies. Under SSP285, where there are weak climate policies, persistent fossil fuel dependency, and rapid urban expansion, global temperature will be projected to increase by 4–5°C by 2100. These rapid increases in the Earth’s temperature and shift in its climate system will lead to the occurrence of new pests and pest resurgence. Therefore, understanding how tef head smudge disease and tef crop respond to the current and future climate change is crucial to designing effective management strategies.

Species distribution models (SDMs) are used to quantify the response of several species to climate change under several climate change scenarios [18]. However, the maximum entropy (MaxEnt) species distribution model is the most commonly employed model for quantifying the response of several species to climate change due to its high predictive performance and least sensitivity to small sample sizes [19]. This model has been employed for modeling the habitat suitability and distribution potential of *Aloe ankoberensis* and *Aloe debrana* in Ethiopia [20]. Moreover, it is used in modeling tef head smudge disease in the Western Amhara Region, Ethiopia [11]. Therefore, quantifying the spatiotemporal intersection of tef and tef head smudge disease is crucial to reduce yield loss, delineate tef seed production sites, conservation of genetic resources, and deploy effective management strategies. Therefore, this study aims to: (1) quantify the current geographic distribution of tef and tef head smudge disease in the Western Amhara Region (2) Quantify the projected geographic distribution of tef and tef head smudge disease under SSP 245 and SSP 285 climate change scenarios by 2050 and 2070 (3) identify the common areas of tef head smudge disease and tef (geographic intersection) under the current and future climate change scenarios.

## 2. Materials and methods

### 2.1. Description of the study areas

The locations of the surveyed areas lay between 36^o^73’ to 38^o^24’ E longitudes, and 10^o^19’ and 11^o^89’ N latitudes. The altitudes of these areas ranged from 1744 to 2705 m.a.s.l. The study area has an average minimum and maximum temperature of 8.1 to 28.4 °C, respectively, whereas the rainfall ranges from 507.3 to 1379.8 mm in a unimodal pattern. The soil type was heavy to light vertisols, and brown to red nitosols. Major crops grown in survey target areas were cereals, legumes, and oil crops.

**Fig 1:**
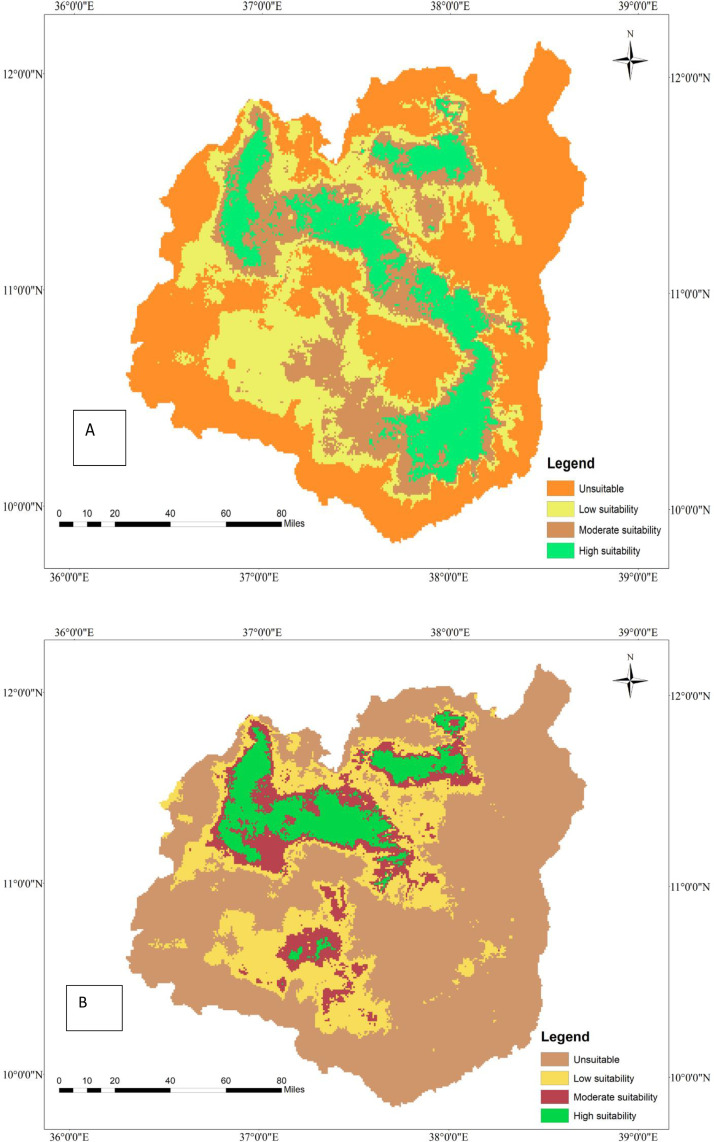
Current geographic distribution of tef (A) and tef head smudge disease (B) in the Western Amhara region, Ethiopia.

### 2.2. Species occurrence data

Tef head smudge disease and tef crop species occurrence data were obtained through conducting a survey. The survey was conducted in the Western Amhara Region, Ethiopia, during the 2017 cropping season at 5–10 km intervals. During the survey, a total of 191 tef fields and 93 tef head-smudge-infested fields, along with their geographic coordinates, were recorded. The data of tef head smudge disease-infested fields (93) and tef crop fields (191) were encoded and organized in an Excel file as per the requirements of the MaxEnt software, as described in Phillips [21] for model building. The maximum entropy model (MaxEnt), which is known to be insensitive to small sample sizes, was used for modeling.

### 2.3 Environmental layers data

A total of 32 environmental variables were used to model the geographic distribution of tef head smudge disease and tef crop in the Western Amhara Region, Ethiopia (Table 1). Environmental variables were downloaded from the WorldClim dataset (www.worldclim.org) at 1 km^2^ resolution. Tef head smudge disease mainly occurs between September and December. Therefore, only the September to December wind speed and solar radiation data were downloaded from the WorldClim dataset. The second-generation Euro-Mediterranean Centre on Climate Change Earth System Model (CMCC-ESM2) from the CMIP6 general circulation models (GCMs) was used for future prediction. The CMCC-ESM2 climate projection model had good performance for the Ethiopian environment [22]. Therefore, the future climate prediction for the years 2050 (2041–2060) and 2070 (2061–2080) for moderate (SSP 245) and high emission scenarios (SSP285) was downloaded from the WorldClim website.

**Table 1:** Environmental variables used for Maximum Entropy (MaxEnt) species distribution modeling.

No	Environmental variables	Unit
1	BIO1 (Annual mean temperature)	Degrees Celsius
2	BIO2 (Mean diurnal range)	Degrees Celsius
3	BIO3 (Isothermally)	Degrees Celsius
4	BIO4 (Temperature seasonality)	Degrees Celsius
5	BIO5 (Max temperature of warmest month)	Degrees Celsius
6	BIO6 (Min temperature of the coldest month)	Degrees Celsius
7	BIO7 (Temperature annual range)	Degrees Celsius
8	BIO8 (Mean temperature of wettest quarter)	Degrees Celsius
9	BIO9 (Mean temperature of driest quarter)	Degrees Celsius
10	BIO10 (Mean temperature of warmest quarter)	Degrees Celsius
11	BIO11 (Mean temperature of coldest quarter)	Degrees Celsius
12	BIO12 (Annual precipitation)	Millimeters
13	BIO13 (Precipitation of wettest month)	Millimeters
14	BIO14 (Precipitation of the driest month)	Millimeters
15	BIO15 (Precipitation seasonality)	Percentage/fraction
16	BIO16 (Precipitation of wettest quarter)	Millimeters
17	BIO17 (Precipitation of driest quarter)	Millimeters
18	BIO18 (Precipitation of warmest quarter)	Millimeters
19	BIO19 (Precipitation of coldest quarter)	Millimeters
20	Rad 9 (Solar radiation of September)	Watts per square meter (W/m²)
21	Rad 10 (Solar radiation of October)	Watts per square meter (W/m²)
22	Rad 11 (Solar radiation of November)	Watts per square meter (W/m²)
23	Rad 12 (Solar radiation of December)	Watts per square meter (W/m²)
24	Win 9 (wind speed of September)	Meters per second (m/s)
25	Win 10 (wind speed of October)	Meters per second (m/s)
26	Win 11 (wind speed of November)	Meters per second (m/s)
27	win12 (wind speed of December)	Meters per second (m/s)
28	Hum 9 (humidity of September)	Percentage (%)
29	Hum 10 (humidity of October)	Percentage (%)
30	Hum 11 (humidity of November)	Percentage (%)
31	Hum 12 (humidity of December)	Percentage (%)
32	Elevation	Meter

Environmental layers of the Western Amhara Region, Ethiopia, were clipped using ArcGIS from the global environmental layers as described in Khwarahm [23] and Phillips [21]. Afterward, the clipped environmental layers were ensured to have the same pixel size and coordinate reference system, and exported as ASCII files to ensure compatibility with MaxEnt software [21].

### 2.4. Selection of environmental variables

The Pearson correlation coefficient was used to account for multicollinearity between environmental variables, and variables with a correlation coefficient >0.8 were excluded to minimize the effect of multicollinearity and model over-fitting [24,25]. Therefore, among 32 environmental variables, 21 environmental variables below this threshold were used to model the current and future distributions of tef head smudge disease in the Western Amhara region (Table 2).

**Table 2:** Lists of environmental variables selected based on the Pearson correlation coefficient.

No	Selected environmental variables	Unit
1	BIO1 (Annual mean temperature)	Degrees Celsius
2	BIO2 (Mean diurnal range)	Degrees Celsius
3	BIO3 (Isothermally)	Degrees Celsius
4	BIO4 (Temperature seasonality)	Degrees Celsius
5	BIO7 (Temperature annual range)	Degrees Celsius
6	BIO8 (Mean temperature of wettest quarter)	Degrees Celsius
7	BIO10 (Mean temperature of warmest quarter)	Degrees Celsius
8	BIO11 (Mean temperature of coldest quarter)	Degrees Celsius
9	BIO12 (Annual precipitation)	Millimeters
10	BIO13 (Precipitation of wettest month)	Millimeters
11	BIO15 (Precipitation seasonality)	Percentage/fraction
12	BIO16 (Precipitation of wettest quarter)	Millimeters
13	BIO18 (Precipitation of warmest quarter)	Millimeters
14	BIO19 (Precipitation of coldest quarter)	Millimeters
15	Rad 10 (Solar radiation of October)	Watts per square meter (W/m²)
16	Rad 11 (Solar radiation of November)	Watts per square meter (W/m²)
17	Rad 12 (Solar radiation of December)	Watts per square meter (W/m²)
18	Win 10 (wind speed of October)	Meters per second (m/s)
19	Win 11 (wind speed of November)	Meters per second (m/s)
20	Hum 10 (humidity of October)	Percentage (%)
21	Hum 11 (humidity of November)	Percentage (%)

### 2.5. Species distribution modeling and model performance evaluation

Maximum entropy (MaxEnt) species distribution models were used for modeling the geographic distribution of tef head smudge disease and tef crop under the current and future climate change scenarios. The MaxEnt species distribution model is based on occurrence data points and predictor variables (21 environmental variables). Bioclimatic and non-climatic variables were imported as environmental layers into MaxEnt software version 3.4.4 [26], whereas occurrence data points were imported into the samples section of the MaxEnt software.

70% of the species occurrences data were used for training, whereas 30% of the data were used for evaluating the accuracy of the model [27]. A bootstrapping replication approach with ten times replication was used. The maximum number of background points and iterations of the MaxEnt software was set to 10,000 and 500, respectively. A jackknife test was used to measure the importance of variables, and a logistic output format was employed to produce a continuous habitat suitability map. The suitability map was then classified into discrete categories to delineate areas based on their suitability levels to the disease. The suitability levels were reclassified into the following classes: 0–0.05 (unsuitable), 0.05–0.37 (low suitability), 0.37–0.69 (moderate suitability) and 0.69–1.0 (high suitability) as described in Birhanie and Dires [11]. The geographic interaction of the tef crop and tef head smudge disease for the current and future climate change scenarios was determined using the intersection function of ArcGIS. The performance of the model was evaluated using the area under the receiver operating characteristic curve (AUC) as described in Mirhashemi et al. [28].

## 3. Results

### 3.1. MaxEnt model performance in predicting the current geographic distribution of tef

The MaxEnt model was good in predicting the current geographic distribution of tef. The model performance metrics, the Area under the Receiver Operating Characteristic Curve (AUC), have achieved a good prediction potential for the tef crop. The Area under the Receiver Operating Characteristic Curve (AUC), the performance metric of the MaxEnt model, had 89.8%% performance in predicting the geographic distribution of tef (Table 3). This high AUC value underscores the model’s strong predictive accuracy. The performance of the models is typically evaluated based on their Area under the Curve (AUC) values, which are classified as weak (0.5–0.7), good (0.7–0.9), or excellent (greater than 0.9).

**Table 3:** Performance metrics of the MaxEnt model (AUC) for the current, 2050, and 2070 time periods under SSP245 and 285 climate change scenarios.

Time period	Climate change scenario	Area under the Receiver Operating Characteristic Curve (AUC) for tef and tef head smudge disease	
		AUC of tef	AUC of the tef head smudge
2050	SSP245	89.9%	93.5%
	SSP285	89.3%	93.3%
2070	SSP245	89.5%	93.6%
	Ssp285	90.5%	93.5%
Current		89.8%	93.4%

### 3.2. MaxEnt model performance in predicting the current geographic distribution of tef head smudge disease

The MaxEnt model was excellent in predicting the current geographic distribution of tef head smudge disease. According to the performance metrics of the MaxEnt model, the model has achieved an excellent prediction potential for tef head smudge disease. The Area under the Receiver Operating Characteristic Curve (AUC), the performance metrics of the MaxEnt model, had 93.4% performance in predicting the geographic distribution of tef head smudge disease (Table 3).

### 3.3. MaxEnt model performance in predicting the future (2050 and 2070) geographic distribution of tef under SSP 245 and 285 climate change scenarios

The Maximum Entropy (MaxEnt) model has demonstrated an excellent predictive accuracy in forecasting the distribution of tef crop by 2050 under the SSP245 climate change scenario. The model has achieved a 90% accuracy in predicting the distribution of tef crop by 2050 under a SSP245 climate change scenario (Table 3). According to the performance metrics of the MaxEnt model, AUC, the model had 89.3%, 89.5% and 90.5% prediction performance for the tef crop for the 2050 extreme, 2070 moderate, and 2070 extreme climate change scenarios, respectively (Table 3). This indicates that the model provides reliable insights in predicting the geographic distribution of tef, making it an indispensable tool for researchers, policymakers, and agricultural stakeholders.

### 3.4. MaxEnt model performance in predicting the future geographic distribution of tef head smudge disease under SSP245 and 285 climate change scenarios

The MaxEnt species distribution model was excellent in predicting the geographic distribution of the tef head smudge disease by 2050 and 2070 under SSP245 and 285 climate change scenarios. The Area under the Receiver Operating Characteristic Curve (AUC), the performance metrics of the MaxEnt model, had 93.5%, 93.3%, 93.6%, 93.5%, and 93.4% performance in predicting the geographic distribution of tef head smudge for the 2050s moderate, 2050s extreme, 2070s moderate, and 2070s extreme climate change scenarios, respectively (Table 3).

### 3.5. Percent contribution of environmental variables to tef crop and tef head smudge disease prediction for the current climate change scenario

The jackknife test indicates that the distribution of tef was mainly influenced by the annual precipitation (BIO12), mean diurnal range (BIO2), and humidity of October (Hum 10). They contribute 20.2%, 11.3% and 8.9% respectively to the MaxEnt model (Table 4). Likewise, mean diurnal range (BIO2), temperature annual range (BIO7), and solar radiation of November (Rad 11) were found to be the top contributors to the MaxEnt model in predicting the current geographic distribution of tef head smudge disease. They contribute 39%, 27.1% and 7.3% respectively for the model (Table 4).

**Table 4:** Contribution of environmental variables to the model for tef crop and Tef head smudge disease.

No	Environmental variables	Percent Contribution (%)	
		Tef	Tef head smudge disease
1	BIO1 (Annual mean temperature)	2.6	1.3
2	BIO2 (Mean diurnal range)	11.3	39
3	BIO3 (Isothermally)	2.4	3.6
4	BIO4 (Temperature seasonality)	4.1	3.9
5	BIO7 (Temperature annual range)	3.7	27.1
6	BIO8 (Mean temperature of wettest quarter)	0.2	0.1
7	BIO10 (Mean temperature of warmest quarter)	3.5	1.2
8	BIO11 (Mean temperature of coldest quarter)	3.7	0.4
9	BIO12 (Annual precipitation)	20.2	0.9
10	BIO13 (Precipitation of wettest month)	0.3	0.9
11	BIO15 (Precipitation seasonality)	7.6	4
12	BIO16 (Precipitation of wettest quarter)	2.3	1.6
13	BIO18 (Precipitation of warmest quarter)	3.1	1.6
14	BIO19 (Precipitation of coldest quarter)	4.7	2.4
15	Rad 10 (Solar radiation of October)	4.8	1.8
16	Rad 11 (Solar radiation of November)	1.3	7.3
17	Rad 12 (Solar radiation of December)	0.6	1
18	Win 10 (wind speed of October)	0.4	0.1
19	Win 11 (wind speed of November)	4.9	0.3
20	Hum 10 (humidity of October)	8.9	0.4
21	Hum 11 (humidity of November)	0.1	1.2

### 3.6. Contribution of environmental variables for predicting the future (2050 and 2070) geographic distribution of tef under SSP245 and 285 climate scenarios

BIO12 (Annual precipitation), BIO2 (Mean diurnal range), Hum 11 (humidity of November), and Rad 10 (Solar radiation of October) are the top contributing environmental variables for the moderate climate change scenario (SSP245) by 2050. They have contributed 20.3%. 10.4%, 9.6% and 7.4% respectively, to the model. BIO12 (Annual precipitation), Hum 11 (humidity of November), BIO2 (Mean diurnal range), and BIO15 (Precipitation seasonality) are the top contributors to the SSP 285 climate change scenario by 2050. They contributed 19.5%, 9.8%, 8.4% and 7.5% respectively (Table 5).

**Table 5:** Percent contribution of environmental variables by 2050 and 2070 under moderate and extreme climate change scenarios for projected tef distribution prediction.

No	Environmental variables	Percent Contribution (%)			
		2050		2070	
		SSP245	SSP285	SSP245	SSP285
1	BIO1 (Annual mean temperature)	2.8	2	2.9	2.6
2	BIO2 (Mean diurnal range)	10.4	8.4	8.9	12
3	BIO3 (Isothermally)	4.2	1.9	4.7	2
4	BIO4 (Temperature seasonality)	3.4	4.1	3.4	4.9
5	BIO7 (Temperature annual range)	4.3	6.3	5	3.6
6	BIO8 (Mean temperature of wettest quarter)	0.5	0.8	1.7	13.8
7	BIO10 (Mean temperature of warmest quarter)	4.5	2.8	8.3	2.4
8	BIO11 (Mean temperature of coldest quarter)	2	5.6	5.3	2.2
9	BIO12 (Annual precipitation)	20.3	19.5	19.1	19.1
10	BIO13 (Precipitation of wettest month)	2	0.6	1.3	0.1
11	BIO15 (Precipitation seasonality)	5.8	7.5	7.4	8.1
12	BIO16 (Precipitation of wettest quarter)	1.8	2	2.4	2
13	BIO18 (Precipitation of warmest quarter)	2.6	1.6	3.3	1.8
14	BIO19 (Precipitation of coldest quarter)	3.7	4.4	2.2	6.3
15	Rad 10 (Solar radiation of October)	7.4	7.4	3.5	6.3
16	Rad 11 (Solar radiation of November)	2.7	2.7	1.8	2.6
17	Rad 12 (Solar radiation of December)	2.4	2.6	1.2	2.4
18	Win 10 (wind speed of October)	2.6	2.8	1.9	0.3
19	Win 11 (wind speed of November)	4.9	4	5.6	4.1
20	Hum 10 (humidity of October)	2.1	3.2	4.2	0.1
21	Hum 11 (humidity of November)	9.6	9.8	5.9	3.3

According to the jackknife test, BIO12 (Annual precipitation), BIO2 (Mean diurnal range), BIO10 (Mean temperature of warmest quarter), and BIO15 (Precipitation seasonality) are the top contributors for the 2070 SSP245 climate change scenario. They have contributed 19.1%, 8.9%, 8.3% and 7.4% respectively for the model. The jackknife test also found that BIO12 (Annual precipitation), BIO8 (Mean temperature of wettest quarter), BIO2 (Mean diurnal range), and BIO15 (Precipitation seasonality) are the top contributors to the SSP 285 by 2070, contributing 19.1%, 13.8%, 12% and 8.1% respectively (Table 5).

### 3.7. Contribution of environmental variables to tef head smudge disease distribution prediction by 2050 and 2070 under SSP245 and 285 climate scenarios

According to the jackknife test, BIO2 (Mean diurnal range), BIO7 (Temperature annual range), and Rad 11 (Solar radiation of November) are the top contributors of the MaxEnt model by 2050 under SSP245and SSP285. They have contributed 37.7%, 25.6% and 8.2% respectively, by 2050 under SSP245. However, they have contributed 36.4%, 24.5% and 8.5% respectively by 2050 under SSP285. Similarly, BIO2 (36.4%), BIO7 (24.5%%), and Rad 11 (8.5%) are the top contributors of the MaxEnt model by 2070 under the SSP285 climate change scenario. However, BIO2 (40.8%), BIO7 (28.6%), and BIO15 (Precipitation seasonality, 5.8%) were found to be the top contributors to the MaxEnt model by 2070 under the SSP245 climate change scenario (Table 6).

**Table 6:** Environmental variables’ percent contribution to the tef head smudge disease distribution predictions by 2050 and 2070 under SSP245 and SSP285 scenarios.

No	Environmental variables	Percent Contribution (%)			
		2050		2070	
		SSP245	SSP285	SSP245	SSP285
1	BIO1 (Annual mean temperature)	1.3	1.2	1.5	1.7
2	BIO2 (Mean diurnal range)	37.7	36.4	40.8	36.4
3	BIO3 (Isothermally)	3.7	4.7	3.6	4.7
4	BIO4 (Temperature seasonality)	4.9	5.3	3.1	5.3
5	BIO7 (Temperature annual range)	25.6	24.5	28.6	24.5
6	BIO8 (Mean temperature of wettest quarter)	0.1	0.4	0.2	0.4
7	BIO10 (Mean temperature of warmest quarter)	1.4	2.2	0.1	1.2
8	BIO11 (Mean temperature of coldest quarter)	0.7	0.2	0.5	0.2
9	BIO12 (Annual precipitation)	0.2	0.5	0.7	0.5
10	BIO13 (Precipitation of wettest month)	1.8	1	1.1	1
11	BIO15 (Precipitation seasonality)	4.6	3.2	5.8	3.2
12	BIO16 (Precipitation of wettest quarter)	2.3	2.6	1.7	2.6
13	BIO18 (Precipitation of warmest quarter)	0.7	1.3	1.1	1.3
14	BIO19 (Precipitation of coldest quarter)	2.6	1.4	2.6	1.4
15	Rad 10 (Solar radiation of October)	0.9	1.3	2.4	1.3
16	Rad 11 (Solar radiation of November)	8.2	8.5	2.8	8.5
17	Rad 12 (Solar radiation of December)	0.4	1.9	0.3	1.9
18	Win 10 (wind speed of October)	0.2	0.7	0.4	0.7
19	Win 11 (wind speed of November)	0.5	0.5	0.4	0.5
20	Hum 10 (humidity of October)	1	0.7	0.3	0.7
21	Hum 11 (humidity of November)	1.2	1.5	2	2

### 3.8. Current geographic distribution of tef (*Eragrostis tef*) and tef head smudge disease

Tef is widely cultivated in the Western Amhara Region in four administrative zones, namely East Gojjam, West Gojjam, Awi, and South Gonder zones (Fig 1A). Achefer, Adet, and Merawi districts of the West Gojjam zone take the lion’s share of tef production, whereas a significant amount of tef cultivation occurs in Farta, Dera, and Simada districts of the South Gonder zone. Moreover, East Gojjam and Awi Zones contribute to the region’s overall production capacity. The MaxEnt model estimated that approximately 17,179 hectares of land in the Region are under tef cultivation (Table 7). Although tef is serving as a food security crop in the region, tef head smudge disease is threatening its production and productivity in several districts of the region (Fig 1B). The MaxEnt model predicts that tef head smudge disease is prevalent in several districts of the Western Amhara Region, specifically in Achefer, Merawi, Adet, Farta, Dera, and Estie. Moreover, this devastating fungal disease is predicted to occur in Machakel, Gozamen, Jabi Tenhan, and Hulet Eju Enesie districts of East Gojjam zone and similar agroecologies. According to the MaxEnt model, tef head smudge disease infests about 63.75% (10951 ha) of tef-producing area in the Western Amhara Region (Table 7)

**Table 7:** Percent cover of tef and tef head smudge disease in the Western Amhara Region, Ethiopia.

	Current	2050		2070	
		SSP245	SSP285	SSP245	SSP285
Total area (ha)	52373	52373	52373	52373	52373
Tef producing area (ha)	17179	12089	21266	17674	10459
Percent cover of tef-producing area (%)	33	23.10	40.60	33.70	19.97
Smudge infested area (ha)	10951	6361	18812	8193	7486
Percent cover of tef head smudge (%)	63.75	52.62	88.46	46.36	71.57

**Fig 2:**
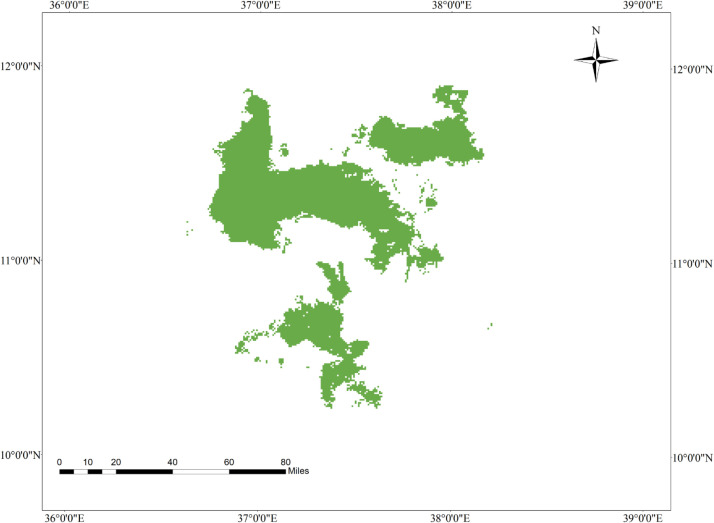
Geographic interaction of tef head smudge disease and its host crop in the Western Amhara Region, Ethiopia.

### 3.9. Current geographic interaction of tef (*Eragrostis tef*) and tef head smudge disease in the Western Amhara Region, Ethiopia

The MaxEnt model reveals that there is a spatial overlap between tef cultivation and tef head smudge disease in the districts of Achefer, Merawi, and Adet, located in the West Gojjam Zone. Moreover, tef head smudge disease has a shared geographic distribution with tef crop across Jabi Tenhan, Machakel, Gozamen, Quarit, and Burie Wonberma districts (Fig 2). Similarly, Dera, Estie, and Farta districts of South Gonder Zone have a spatial co-occurrence of tef (*Eragrostis tef*) and tef head smudge disease. According to the MaxEnt model, 9659 ha of land is found to be suitable for both tef and tef head smudge disease (Table 8).

**Table 8:** Intersection area of tef and its host crop in the Western Amhara Region, Ethiopia.

	Current	2050		2070	
		SSP245	SSP285	SSP245	SSP285
Tef producing area (ha)	17179	12089	21266	17674	10459
Smudge infested area (ha)	10951	6361	18812	8193	7486
Intersection area (ha)	9659	4679	15846	4679	3334

**Fig 3:**
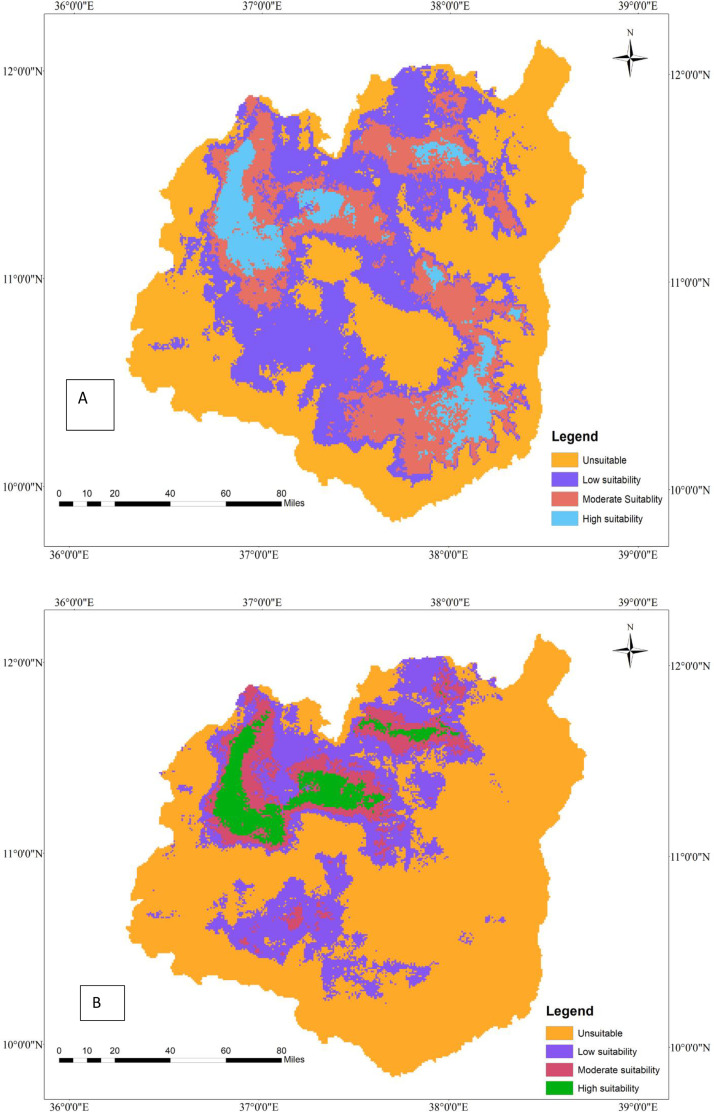
Geographic distribution of tef (A) and tef head smudge disease (B) by 2050 under a moderate SSP245 climate change scenario in the Western Amhara Region, Ethiopia.

### 3.10. Projected geographic distribution of tef and tef head smudge disease by 2050 under SSP245 climate scenarios in the Western Amhara Region, Ethiopia

According to the MaxEnt model, tef will lose some of its geographic range by 2050 under the SSP245 climate change scenario as compared to the current climate change scenario (Table 7). The MaxEnt model predicted that approximately 12,089 hectares of land would be suitable for tef cultivation by 2050 under the SSP245 climate change scenario. However, the predicted geographic range of tef by 2050 under the SSP245 climate scenario will be 7% less than the current cultivation area (17179 ha). Although tef loses its geographic range by 2050 under the SSP245 climate change scenario, it will be cultivated in Achefer, Dangla, Banja, Fagta Lekoma, Adet, Dera, Estie, and Farta districts. Moreover, this precious orphan crop will be cultivated in Shebel Berenta, Baso Liben, Gozamen, Enemay, and any other districts of the East Gojjam zone (Fig 3A). Although tef will be predicted to be cultivated in several areas of the Western Amhara region, its production will be constrained by tef head smudge disease by 2050 under the SSP245 climate change scenario.

**Fig 4:**
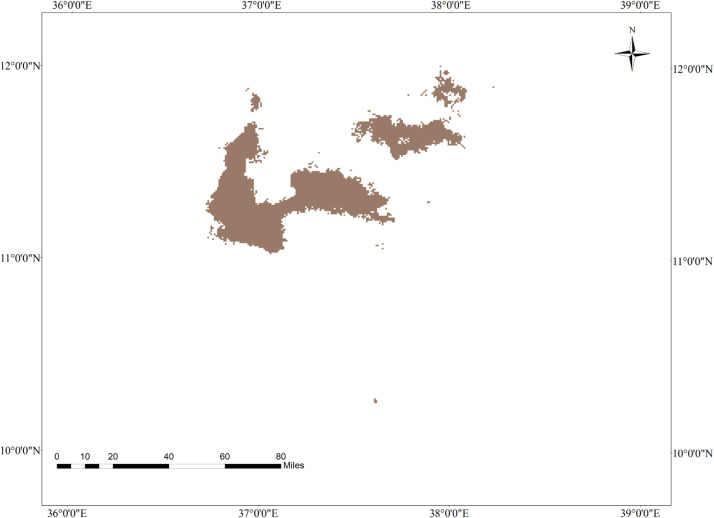
Geographic interaction of tef head smudge disease and its host crop by 2050 under the SSP245 climate change scenario in the Western Amhara Region, Ethiopia.

The distribution of tef head smudge disease will decrease by 2050 under the SSP245 climate change scenario as compared to the current climate change scenario. The MaxEnt model prediction indicated that the geographic range of tef head smudge disease will contract by 0.58% (6,361 hectares) from its current distribution of 10,951 hectares. Therefore, the tef head smudge will lose Gozamen, Machakel, Denbecha, Quarit, and Hulet Eju Enebsie districts, where it used to be present under the current climate change scenario. However, it will be found in Merawi, Achefer, Adet, Dangla, Dera, Estie, and Farta districts (Fig 3B).

### 3.11. Geographic interaction of tef head smudge disease and tef by 2050 under the SSP245 climate change scenario in the Western Amhara Region, Ethiopia

The MaxEnt model forecasts that the spatial interaction between tef and tef head smudge disease will decline by 2050 under the SSP245ate climate change scenario, as compared to the current climatic conditions. However, tef and tef head smudge disease will co-occur on 4679 ha of land by 2050 under the SSP245 climate change scenario (Table 8). The tef head smudge and tef crop will geographically overlap in Achefer, Adet, Merawi, Bahir Dar, Dera, and Farta districts of the Western Amhara Region. However, the geographic overlap of tef and tef head smudge disease will diminish by 0.48% by 2050 under the SSP245 climate change scenario, as compared to the current situation, and some areas will lose their geographic overlap of tef and tef head smudge disease. According to the model, tef head smudge disease and its host crop will lose their geographic overlap in Machakel, Gozamen, Jab Tenhan, Quarit, and Hulet Eju Enesie districts by 2050 under the SSP245 climate change scenario as compared to the current climate change scenario (Fig 4).

**Fig 5:**
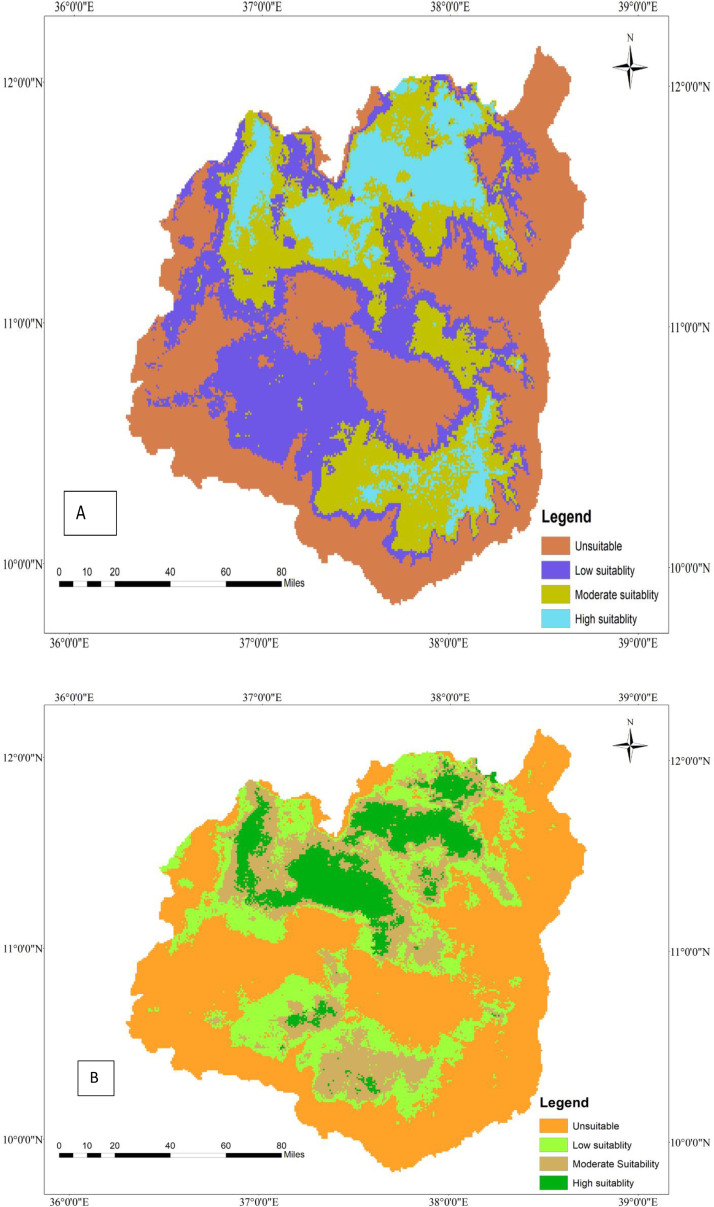
Geographic distribution of tef (A) and tef head smudge disease (B) by 2050 under the SSP285 climate change scenario in the Western Amhara Region.

### 3.12. Projected geographic distribution of tef and tef head smudge disease by 2050 under the SSP285 climate scenarios

The MaxEnt model predicts that the geographic range of tef will extend to new areas by 2050 under the SSP285 climate change scenario, as compared to 2050 under the SSP245 climate change scenario. The MaxEnt model predicted that the distribution of tef will expand in Simada, Estie, Dera, Fogera, and Farta districts of the south Gonder Zone, whereas the distribution of the crop will have an insignificant change in East Gojjam and West Gojjam zones (Fig 5A). Therefore, a substantial proportion of the Western Amhara Region (approximately 40.6%) will be suitable for tef cultivation. The distribution of tef by 2050 under the SSP285 climate change scenario will exceed the 2050 SSP245 and the current distribution of tef by 17.5% and 7.6% respectively (Table 7).

**Fig 6:**
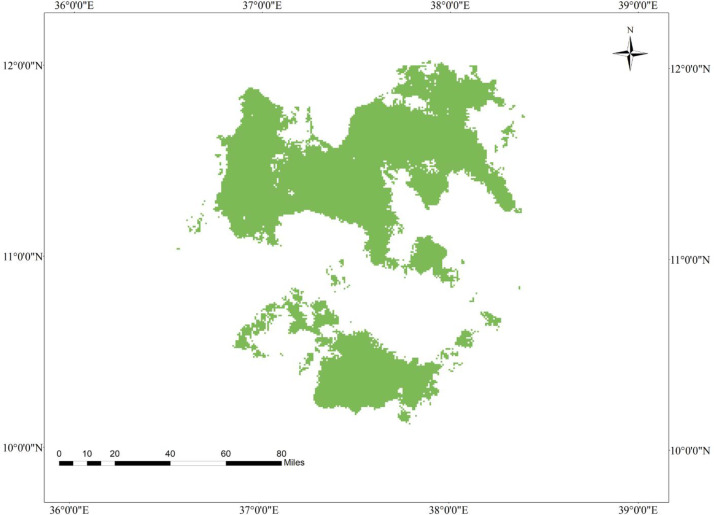
Geographic intersection of tef and tef head smudge disease by 2050 under the SSP285 climate change scenario in the Western Amhara Region.

The geographic distribution of tef head smudge disease will expand by 2050 under the SSP285 climate change scenario, as compared to the 2050 moderate climate change scenario. The MaxEnt model predicted that tef head smudge disease is expected to expand in Machakel, Jabi Tehinan, Gozamen, Simada, and Estie districts of the region by 2050 under SSP285 (Fig 5B). Furthermore, the model predicts that a total of approximately 18,812 hectares of land in the Western Amhara Region will become suitable for the development of tef head smudge disease (Table 7).

### 3.13. Geographic interaction of tef and tef head smudge disease by 2050 under the SSP285 climate change scenario in the Western Amhara Region

The geographic interaction of tef and tef head smudge disease will increase drastically by 2050 under the SSP285 climate change scenario, as compared to the 2050 moderate climate change scenario. The geographic interaction of tef head smudge disease and its host crop will expand to Gozamen, Machakel, Jabi Tenhan, Enarj Enawga, and Simada districts by 2050 under the SSP285 climate change scenario (Fig 6). The MaxEnt model predicts that the Western Amhara region will largely become favorable for the development of tef head smudge disease, with a substantial geographic overlap of approximately 15,846 hectares between the disease and its host crop (Table 8).

**Fig 7:**
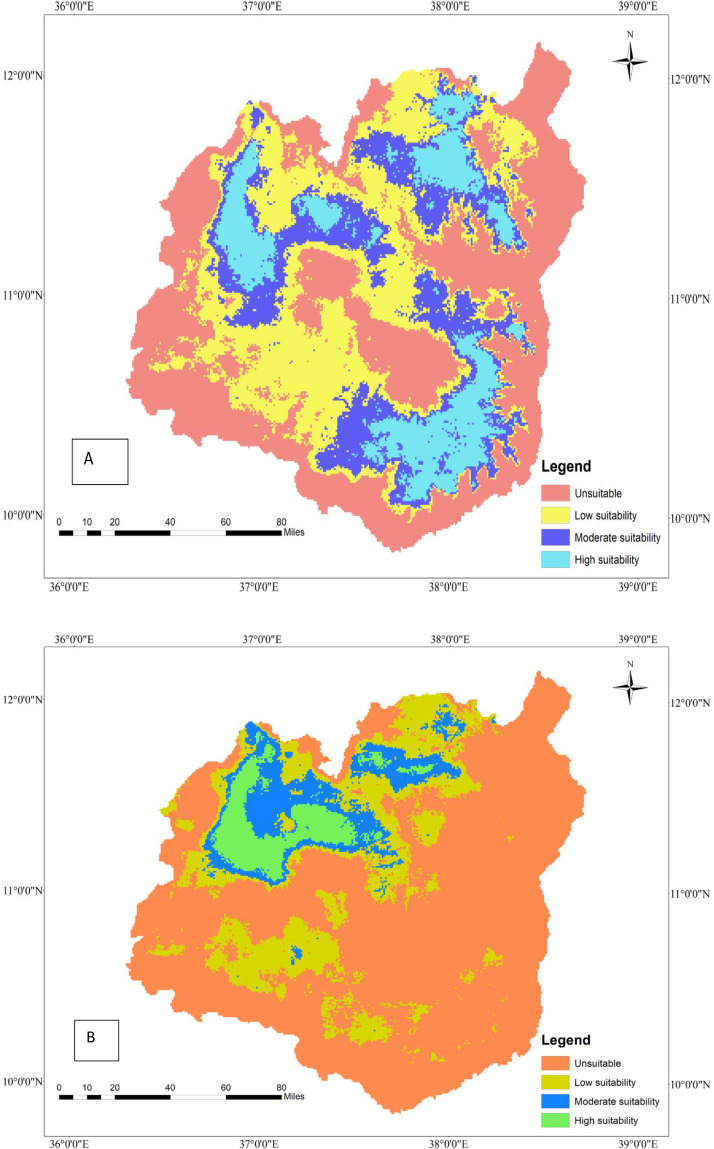
Geographic distribution of tef (A) and tef head smudge disease (B) by 2070 under the SSP245 climate change scenario in the Western Amhara Region.

### 3.14. Geographic distribution of tef and tef head smudge disease by 2070 under the SSP245 climate change scenario in the Western Amhara Region, Ethiopia

The geographic distribution of tef is restricted to the midland areas of East Gojjam, West Gojjam, Awi, and South Gonder Zones under the current climatic conditions. However, its geographic distribution will expand to the highlands of the South Gonder zone by 2070 under the SSP245 climate change scenario (Fig 7A). The MaxEnt model forecasts that approximately 33.7% (17,674 hectares) of the Western Amhara Region will have favorable conditions for tef cultivation. Although 33.7% of the study area will be suitable for tef cultivation, the production of tef will be constrained by tef head smudge disease across its geographic range. The MaxEnt model predicts that out of the 17,674 hectares of land deemed suitable for tef cultivation, approximately 46.36% (8,191 hectares) is expected to be prone to the development of tef head smudge disease (Table 7). Therefore, tef head smudge disease will be found in Dangla, Fagta Lekoma, Merawi, Bahir Dar, Adet, and some pocket areas of Jabi Tenhan districts. Moreover, Dera, Este, Farta, and some areas of the Fogera district will be conducive to the tef head smudge disease (Fig 7B).

**Fig 8:**
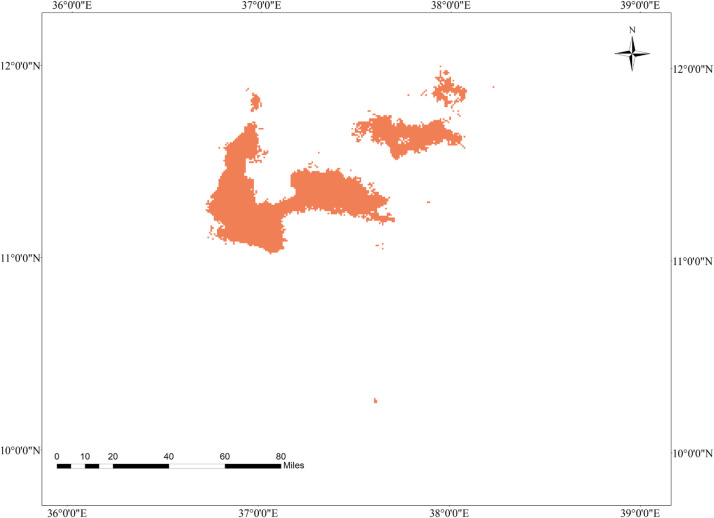
Geographic intersection of tef and tef head smudge disease by 2070 under the SSP245 change scenario in the Western Amhara Region.

### 3.15. Geographic intersection of tef head smudge disease and its host crop by 2070 under the SSP245 climate change scenario in the Western Amhara Region

The geographic intersection of tef and tef head smudge disease is restricted to West Gojjam, Awi, and South Gonder districts. Tef and tef head smudge disease will highly overlap in the West Gojjam zone, followed by the South Gonder Zone and Awi zones, respectively. Achefer, Merawi, and Adet districts of the West Gojjam zone, Dera, Estie, and Farta districts of the south Gonder zone, and Dangla district of the Awi zone will be the common grounds of tef head smudge disease and its host crop by 2070 under a moderate climate change scenario (Fig 8). The MaxEnt model projects that 4,679 hectares of land will have a spatial overlap between tef cultivation and tef head smudge disease under by 2070 under the SSP245 climate change scenario (Table 8).

**Fig 9:**
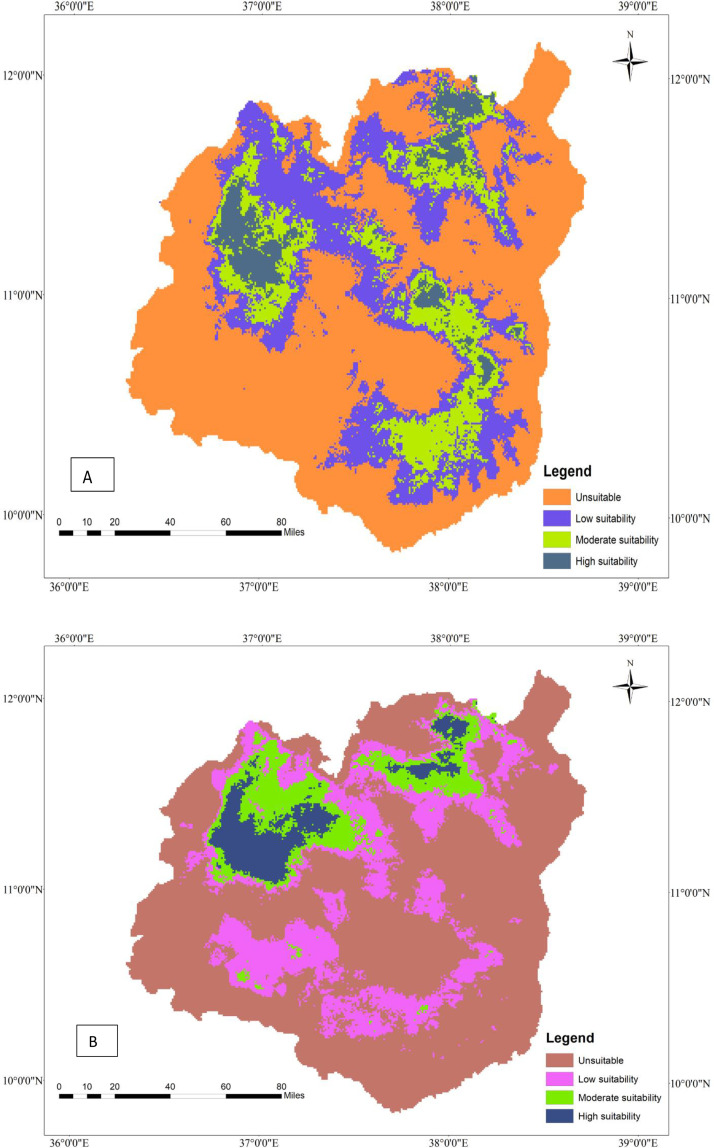
Geographic distribution of tef (A) and tef head smudge disease (B) by 2070 under an extreme climate change scenario in the Western Amhara Region.

### 3.16. Geographic distribution of tef and tef head smudge disease by 2070 under the SSP285 climate change scenario in the Western Amhara Region, Ethiopia

The MaxEnt model predicts that the geographic distribution of tef is expected to encompass East Gojjam, West Gojjam, Awi, and South Gonder Zones of the Western Amhara Region by 2070 under the SSP285 climate change scenario (Fig 9A). It is predicted to occupy about 10459 ha of land by 2070 under the SSP285 climate change scenario (Table 7). However, 71.57% (7486 ha) of it will be conducive to tef head smudge disease (Table 7). Merawi, Achefer, Bahir Dar, Dangla, Fagta Lekoma, Dera, Estie, and Farta districts will be the hotspots of tef head smudge disease. However, East Gojjam will be free from the disease by 2070 under the SSP285 climate change scenario (Fig 9B).

**Fig 10:**
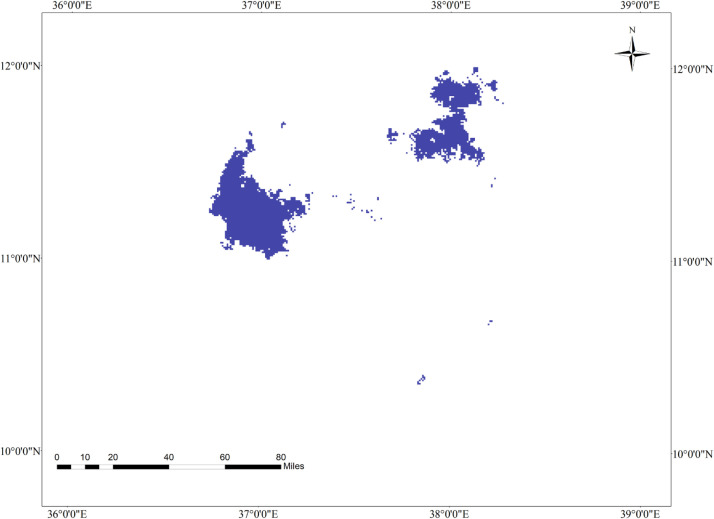
Geographic intersection of tef head smudge disease and tef by 2070 under the SSP285 climate change scenario in the Western Amhara Region.

### 3.17. Geographic interaction of tef and tef head smudge disease by 2070 under the SSP285 climate change scenario in the Western Amhara Region

Tef and tef head smudge disease will overlap in Farta, Estie, Dera, Adet, Merawi, Dangla, Achefer, and Fagta Lekoma districts by 2070 under the SSP285 climate change scenario (Fig 10). The MaxEnt model predicted that approximately 3,334 hectares of land will be subject to the concurrent presence of tef cultivation and tef head smudge disease by 2070 under the SSP285 climate change scenario (Table 8). This suggests that 44.5% (3,334 hectares) of the area projected to be suitable for tef cultivation will also provide optimal growth conditions for tef head smudge disease.

## 4. Discussion

This study provides the first comprehensive analysis of the current and projected geographic distribution of tef head smudge disease and tef under SSP245 and SSP285 climate change scenarios. It also explored the geographic interaction between the tef head smudge disease and tef, providing critical insights for future agricultural adaptation strategies. The MaxEnt species distribution model is the most important method for predicting the potential distribution of a species by producing habitat suitability maps [20,29]. This modeling approach supports conservation efforts by identifying potential habitats for threatened species, facilitating better planning and management. Therefore, the MaxEnt model has gained excellent recognition in delineating seed production zones, conservation of biodiversity, and identification of endangered species in the future [30]. The MaxEnt model has achieved an excellent accuracy in predicting the geographic distribution of tef head smudge disease and tef under the current climate change scenario. Moreover, its projections for 2050 and 2070 have achieved excellent accuracy and offer crucial insights for future agricultural planning and disease management. However, Birhanie and Girmaye [11] found an AUC of 0.85 in predicting the geographic distribution and habitat suitability of tef head smudge disease under the current climate change scenario, and Yebeyen et al. [22] found an AUC of 1 for highland bamboo. Previous studies in Ethiopia have successfully utilized the MaxEnt modeling approach for predicting coffee species distribution, yielding notably high accuracy rates in their forecasts [31]. Similarly, Zewudie et al. [32] found 0.83–0.85 accuracy for both the current and SSP scenarios in predicting the geographic distribution and habitat suitability of tef.

Environmental variables serve as the foundation for understanding how climate change affects species distribution. They help to determine the levels of threats posed by climate change on a species and identify the most critical environmental variables [20,33]. The use of an ample amount of Environmental variables is crucial for effective prediction of a species’ distribution. Therefore, 21 environmental variables were used in this study to improve the accuracy of the MaxEnt model. Among the 21 environmental variables, BIO12, BIO2, and Hum 10 were found to be the top contributors of the MaxEnt model, contributing 20.2%, 11.3% and 8.9% respectively, for the current tef distribution prediction. However, Zewudie et al. [32] found that the current geographic distribution of tef is mainly influenced by the mean temperature of the coldest season (Bio11 and precipitation seasonality (Bio15). The observed disparity in important environmental variables between our study and Zewudie et al. [32] could be due to the differences in the number of environmental variables analyzed. Our study utilized a robust and comprehensive set of approximately 21 variables to ensure accurate prediction, whereas their analysis was limited to 11 environmental variables. This suggests that utilizing an extensive array of environmental variables could significantly enhance the identification of important environmental variables, thereby improving the accuracy and robustness of the model [33]. However, BIO2, BIO7, and Rad 11 were found to be the top contributors to the MaxEnt model in predicting the current geographic distribution of tef head smudge disease. Conversely, Birhanie and Diress [11] found that bio10, bio1, bio9, and BIO7 were important environmental variables for the current prediction of tef head smudge disease. The difference between our studies with other results is due to the use of ample and uncorrelated variables in our study.

BIO12 (Annual precipitation), BIO2 (Mean diurnal range), Hum 11 (humidity of November), BIO8 (Mean temperature of wettest quarter), and BIO15 (Precipitation seasonality) are the top contributors of the MaxEnt model by 2050 and 2070 under moderate and extreme climate change scenarios, contributing 7.4% to 20.3% to the MaxEnt model. However, Zewudie et al. [32] found that the potential distribution of tef under the SSP scenarios was influenced by Bio11 (77.8%–80.4%) and Bio15 (7.3%–9.04%). However, BIO2 (Mean diurnal range), BIO7 (Temperature annual range), and Rad 11 (Solar radiation of November) are the top contributors to tef head smudge prediction by 2050 and 2070 under SSP 4.5 and 8.5. They contribute 5.8% to 37.7% to the model.

Climate change plays a significant role in reducing the area coverage of tef by creating a conducive environment to termite, shoot fly, soil degradation, shattering, and fungal diseases [34]. Therefore, the geographic distribution of tef is anticipated to decrease to 23.1% by 2050 under the SSP245 climate change scenario and will increase to 40.6% by 2050 under the SSP285 climate change scenario, from its currently predicted 33% distribution. Our findings are consistent with Flynn [35], who also reported a decline in tef distribution from early-summer (May-July) to late-summer (August-September). Moreover, Woldeyohannes et al. [36] projected a 2070 increase in tef distribution, primarily in the central highlands, Amhara, and parts of SNNP, which contrasts with our findings, potentially due to their limited consideration of environmental variables.

The distribution of tef will be 33.7% and 19.97% by 2070 under SSP245 and SSP285 climate change scenarios, respectively. Similarly, Zewudie et al. [32] found that the current geographic distribution and habitat suitability of tef decreases in SSP 245 (−4,760 km2) and SSP 285 (−7,345 km2) as compared to the current and SSP 2.6. However, 10951 ha of the Western Amhara region is predicted to be suitable for tef head smudge under the current climate change scenario. This coverage is far below the prediction of Birhanie and dires [11]. They found that 75.64% of the Western Amhara Region is at risk of tef head smudge disease outbreaks. However, our predicted area (10951 ha) is about 21% of the Western Amhara Region. Therefore, our findings depicted that 21% of the western Amhara region and similar agroecologies will be at risk of tef head smudge disease outbreak. The difference in this prediction could be the use of ample environmental variables in our current study.

The distribution of tef head smudge disease is projected to be 6361 ha and 18812 ha by 2050 under moderate and extreme climate change scenarios, respectively. Moreover, the distribution of this disease will be predicted to cover about 8193 and 7486 ha of land by 2070 under moderate and extreme climate change scenarios, respectively. Therefore, major tef-producing areas, such as Adet, Achefer, Macha, Farta, Estie, and Dera, will be impacted by tef head smudge disease under the current and projected climate change scenario. To the best of our knowledge, this is the first projected prediction of the distribution of tef head smudge disease in the Western Amhara Region, Ethiopia.

The coincidence of pests and their hosts in both time and location provides pests with the necessary resources to achieve robust growth and development. However, spatiotemporal separation of the host and its pest can reduce pest pressure without resulting in reduced parasitism [37]. Therefore, tef and tef head smudge disease will have spatial disparity in most of the Western Amhara region under the current and future climate change scenarios, which will result in disease-free conditions. Warner et al. [38] also found a reduced parasitism of Brassica pod midge due to spatial disparity between Brassica pod midge and rape seed. However, the tef head smudge disease and tef overlap to affect approximately 9,659 ha currently, decreasing to 4,679 ha by 2050 under the moderate SSP 245 scenario, and substantially increasing to 15,846 ha by 2050 under the high-emissions SSP 285 scenario. Moreover, tef head smudge and tef will spatially overlap on about 4679 and 3334 ha of land by 2070 under SSP 245 and SSP 285, respectively. This result is in line with Grünig et al. [39], who found a sharp increase in host and pest geographic intersection from the current to RCPs.

## 5. Conclusion

This study investigated the current and projected distribution of tef head smudge disease and tef. Moreover, the study identified the spatiotemporal intersection of tef head smudge and tef across the current, 2050s, and 2070s under the SSP245 and SSP285 climate change scenarios. Such an investigation is vital for researchers and policymakers to deploy monitoring activities and management measures of tef head smudge disease. Moreover, it will help to design conservation strategies of tef and delineate disease-free tef seed-producing sites. This finding underscored that the geographic distribution of tef is projected to decline significantly by 2050 and 2070 under both the SSP245and SSP285 climate change scenarios. Consequently, as the range of tef cultivation diminishes, the geographic distribution of tef head smudge disease is also expected to decrease by 2050 and 2070 under both the SSP 245 and 285 climate change scenarios. Although the geographic distribution of tef and tef head smudge disease is expected to decrease in the future, tef head smudge will remain a big production constraint on predicted intersection areas. In summary, these findings suggest an urgent call for developing appropriate conservation strategies and adaptation measures to tackle the compounded factors contributing to tef habitat loss. Deploying effective tef head smudge disease control strategies, including propiconazole, trifloxystrobin + tebuconazole, and Tebuconazole fungicides, is vital to mitigate the impact of the disease in intersection areas. Moreover, allocating resources to import these essential fungicides (propiconazole, trifloxystrobin + tebuconazole, and Tebuconazole) is crucial to ensure their availability and subsequently reduce yield loss associated with tef head smudge disease in the affected regions.

## Supporting information

S1 File Data. (RAR)
